# Prevalence of Premenstrual Syndrome and Its Impact on Productivity Among Young Female Medical Students

**DOI:** 10.7759/cureus.114116

**Published:** 2026-08-07

**Authors:** Sweta Shrivastava, Khushi Pobari, Tithi H Patel, Anuja Sachapara

**Affiliations:** 1 Department of Obstetrics and Gynaecology, Smt. B. K. Shah Medical College and Research Centre, Sumandeep Vidyapeeth Deemed to be University, Vadodara, IND

**Keywords:** medical students, menstrual health, premenstrual syndrome, productivity, young females

## Abstract

Background

Premenstrual syndrome (PMS) is a common cyclical disorder among reproductive-aged women that adversely affects physical, psychological, and emotional well-being. It is associated with impaired daily functioning, reduced academic performance, and diminished quality of life. Despite its high prevalence, PMS often remains under-recognized and undertreated among young women.

Aim

This study aims to determine the prevalence of premenstrual syndrome among female medical students aged 18-25 years and to evaluate its association with lifestyle factors and its impact on academic productivity.

Methods

A cross-sectional questionnaire-based study was conducted among 250 female medical students aged 18-25 years at Sumandeep Vidyapeeth, Vadodara, Gujarat, India, between May and September 2024.

Results

Among the 250 participants, 188 (75.2%) were identified with PMS. Mood swings (76.4%), abdominal cramps (61.2%), and overthinking (56.0%) were the most frequently reported symptoms. Junk food consumption (70.8%), mental stress (59.6%), and inadequate sleep (49.2%) were common lifestyle factors. PMS was significantly associated with reduced productivity (73.4% versus 14.5%; p<0.001), increased mental stress (p<0.001), and greater analgesic use (p<0.001).

Conclusion

Premenstrual syndrome was highly prevalent among young female medical students and was associated with unhealthy lifestyle factors and impaired academic productivity. Early identification, appropriate counselling, and timely management may reduce symptom burden and improve academic performance and overall quality of life.

## Introduction

Premenstrual syndrome (PMS) is a common cyclic disorder occurring during the late luteal phase of the menstrual cycle and is characterized by physical, emotional, behavioral, and psychological symptoms that begin before menstruation and resolve shortly after its onset [1]. Common symptoms include abdominal pain, backache, headache, bloating, fatigue, breast tenderness, mood swings, anxiety, and irritability, which can adversely affect daily activities, interpersonal relationships, and quality of life [2]. The severe form of PMS is known as premenstrual dysphoric disorder (PMDD).

According to the American College of Obstetricians and Gynecologists (ACOG), PMS is diagnosed when at least one affective and one somatic symptom occur during the five days preceding menstruation in at least three consecutive menstrual cycles and resolve within four days after the onset of menstruation [3]. Globally, PMS affects approximately 20%-40% of menstruating women, while studies from India have reported a prevalence ranging from 14.3% to 74.4% [4,5]. Its etiology is multifactorial and has been associated with lifestyle factors such as inadequate sleep, unhealthy diet, physical inactivity, stress, smoking, alcohol consumption, and caffeine intake [2,6].

Among female medical students, academic stress and demanding schedules may further aggravate PMS symptoms, resulting in reduced concentration, impaired academic performance, and decreased productivity. Despite its significant impact, PMS remains under-recognized, particularly in developing countries where social stigma surrounding menstruation may prevent women from seeking appropriate care [2].

The present study was undertaken to determine the prevalence of premenstrual syndrome (PMS) among female medical students aged 18-25 years, to assess the frequency of common somatic symptoms (including abdominal cramps, bloating, headache, breast tenderness, constipation, and diarrhea) and affective symptoms (including mood swings, anxiety, depression, anger, impaired concentration, and overthinking), to evaluate the association between PMS and selected lifestyle-related factors, and to assess the impact of PMS on self-reported academic productivity and daily functioning.

## Materials and methods

Study design and setting

A cross-sectional questionnaire-based study was conducted among female medical students at Sumandeep Vidyapeeth, Vadodara, Gujarat, India, between May 2024 and September 2024. The study aimed to determine the prevalence of premenstrual syndrome (PMS), identify associated lifestyle factors, and assess its impact on academic productivity among young female medical students.

Study population

Female medical students aged 18-25 years with regular menstrual cycles were eligible to participate. Students who voluntarily agreed to participate and provided written informed consent were enrolled. Students with irregular menstrual cycles, pregnant women, and those unwilling to participate were excluded from the study.

Sample size

The required sample size was calculated using the single population proportion formula for a finite population with the OpenEpi version 3.01 sample size calculator [7], assuming a 95% confidence level and a 5% margin of error. The minimum sample size was estimated to be 250 participants, all of whom were included in the analysis.

Data collection tool and procedure

Data were collected using a predesigned, self-administered, semi-structured questionnaire developed by the investigators specifically for this study (Appendices). The questionnaire was prepared after an extensive review of the published literature on premenstrual syndrome, lifestyle-related risk factors, and the effect of PMS on academic performance and daily functioning. The content of the questionnaire was developed in accordance with the diagnostic criteria for premenstrual syndrome proposed by the ACOG. Prospective daily symptom recording over at least two consecutive menstrual cycles, as recommended by ACOG and the Diagnostic and Statistical Manual of Mental Disorders, Fifth Edition (DSM-5) for confirming PMS and premenstrual dysphoric disorder, was not performed because of the cross-sectional design of the study. No copyrighted or licensed questionnaire was reproduced in its original form; therefore, permission or a license was not required.

The draft questionnaire was reviewed independently by faculty members from the Departments of Obstetrics and Gynecology and Community Medicine to assess its content relevance, clarity, and comprehensiveness.

The final questionnaire consisted of four domains. The first domain collected demographic information and menstrual characteristics, including age, year of study, menstrual regularity, and relevant clinical details. The second domain assessed the presence of premenstrual symptoms, including both affective symptoms (such as anxiety, depression, mood swings, anger, and impaired concentration) and somatic symptoms (such as abdominal cramps, bloating, headache, breast tenderness, constipation, and diarrhea), based on the ACOG diagnostic criteria. The third domain evaluated lifestyle-related factors, including junk food consumption, caffeine intake, smoking or alcohol use, sleep duration, regular exercise, and perceived mental stress. The fourth domain assessed the effect of PMS on productivity using self-reported questions regarding overall productivity, academic performance, concentration during study, attendance at lectures and clinical postings, and routine daily activities. Participants reporting impairment in one or more of these domains were considered to have reduced productivity. Information regarding measures adopted for symptom relief, including analgesic use and other coping strategies, was also collected.

Eligible participants completed the questionnaire anonymously after providing informed consent. Completed questionnaires were checked daily for completeness and consistency before data entry.

Outcome measures

The primary outcome of the study was the prevalence of premenstrual syndrome (PMS) among female medical students. The secondary outcomes included the association of PMS with lifestyle-related factors, its impact on self-reported academic productivity, and the coping strategies adopted for symptom relief. Academic productivity was assessed using self-reported responses regarding overall productivity, academic performance, concentration during study, lecture attendance, clinical postings, and routine daily activities. Participants reporting impairment in one or more of these domains were classified as having reduced productivity. Coping strategies, including analgesic use and other self-management measures, were also recorded. Absenteeism was not evaluated as a separate study outcome.

Statistical analysis

Data were entered into Microsoft Excel 2007 (Microsoft Corp., Redmond, WA, USA) and analyzed using Epi Info statistical software (Centers for Disease Control and Prevention, Atlanta, GA). Continuous variables were summarized as mean ± standard deviation, while categorical variables were expressed as frequencies and percentages. Associations between categorical variables were evaluated using the Chi-square (χ²) test. Variables associated with reduced productivity were further analyzed using multivariable logistic regression to identify independent predictors. Results were expressed as adjusted odds ratios (AORs) with 95% confidence intervals (95% CI), and the significance of individual regression coefficients was assessed using the Wald test. A p-value of <0.05 was considered statistically significant.

Ethical considerations

The study was approved by the Sumandeep Vidyapeeth Institutional Ethics Committee (approval number: SVIEC/ON/Medi/SRP/June/24/103). Written informed consent was obtained from all participants before enrollment. Participation was voluntary, and all information was collected anonymously and kept confidential throughout the study.

## Results

A total of 250 participants were enrolled in this study after fulfilling the inclusion and exclusion criteria. It was found that 188 (75.2%) of the 250 participants were suffering from PMS. The details of the symptoms involved are presented in Table 1.

**Table 1:** Detailed symptomatic presentation of PMS and lifestyle habits of the study cohort PMS: premenstrual syndrome

PMS symptom	Number (%)
Anger issues	123 (49.2)
Anxiety	113 (45.2)
Breast tenderness	59 (23.6)
Bloating	107 (42.8)
Constipation	39 (15.6)
Cramps	153 (61.2)
Diarrhea	26 (10.4)
Depression	63 (25.2)
Headache	79 (31.6)
Lack of concentration	115 (46)
Mood swings	191 (76.4)
Overthinking	140 (56)
None	14 (5.6)
Lifestyle habits	Number (%)
Junk food	177 (70.8)
Addiction (smoking/tobacco/alcohol)	7 (2.8)
Caffeine intake (>2 cups of coffee/tea)	33 (13.2)
Inadequate sleep	123 (49.2)
Regular exercise/yoga	60 (24.0)
Stress	149 (59.6)

Table 2 describes the effect of PMS on the work performance and productivity of the study participants. It was noted that 147 (58.8%) female students had a reduction in overall productivity, and 147 (58.8) female students also used some kind of treatment/intervention for PMS. Eating chocolate/foods was the most common intervention, with 84 (33.6%) participants using it to relieve PMS symptoms. This was closely followed by the use of analgesics by 69 (27.6%).

**Table 2:** Number of participants whose work performance and productivity were affected by PMS and who used interventions for its treatment PMS: premenstrual syndrome

Variable	Number (%)
Overall productivity affected	147 (58.8)
Decreased performance	144 (57.6)
Intervention/treatment (total)	147 (58.8)
Analgesics	69 (27.6)
Hot drinks (tea, coffee, etc.)	50 (20)
Massage	38 (15.2)
Binge eating (chocolate/food)	84 (33.6)
Watching movies/shows/TV	62 (24.8)
Others	27 (10.8)
None	103 (41.2)

Out of the 250 participants, 188 (75.2%) had premenstrual syndrome. Among those with PMS, 138 (73.4%) participants reported affected work productivity, whereas only 9 (14.5%) participants without PMS experienced reduced productivity. The association between PMS and work productivity was found to be statistically significant (χ²=67.58, p<0.001) (Table 3).

**Table 3:** Association between PMS and work productivity among female medical students Values are presented as number (%). Associations between categorical variables were assessed using the Pearson Chi-square (χ²) test (χ²=67.58, p<0.001). PMS: premenstrual syndrome

PMS status	Productivity affected (number (%))	Productivity unaffected (number (%))	Total (number (%))	χ²	p-value
Present	138 (73.4)	50 (26.6)	188 (75.2)	67.58	<0.001
Absent	9 (14.5)	53 (85.5)	62 (24.8)		
Total	147 (58.8)	103 (41.2)	250 (100.0)		

Among participants with premenstrual syndrome, 139 (73.9%) reported consumption of junk food, compared to 38 (61.3%) among those without PMS. The association between PMS and junk food intake showed no statistical significance (χ²≈3.69, p≈0.055) (Table 4).

**Table 4:** Association between PMS and junk food consumption among female medical students Values are presented as n (%). Associations between categorical variables were assessed using the Pearson Chi-square (χ²) test (χ²=3.69, p=0.055). PMS: premenstrual syndrome

PMS status	Junk food consumption present (number (%))	Junk food consumption absent (number (%))	Total (number (%))	χ²	p-value
Present	139 (73.9)	49 (26.1)	188 (75.2)	3.69	0.055
Absent	38 (61.3)	24 (38.7)	62 (24.8)		
Total	177 (70.8)	73 (29.2)	250 (100.0)		

Among the participants with premenstrual syndrome, 124 (66.0%) reported experiencing mental stress, compared to 25 (40.3%) among those without PMS. The association between PMS and mental stress was statistically significant (χ²≈12.80, p<0.001) (Table 5).

**Table 5:** Association between PMS and mental stress among female medical students Values are presented as number (%). Associations between categorical variables were assessed using the Pearson Chi-square (χ²) test (χ²=12.80, p<0.001). PMS: premenstrual syndrome

PMS status	Mental stress present (number (%))	Mental stress absent (number (%))	Total (number (%))	χ²	p-value
Present	124 (66.0)	64 (34.0)	188 (75.2)	12.80	<0.001
Absent	25 (40.3)	37 (59.7)	62 (24.8)		
Total	149 (59.6)	101 (40.4)	250 (100.0)		

Among the 250 participants, 67 (35.6%) with premenstrual syndrome used analgesics, whereas only 2 (3.2%) without PMS reported analgesic use. The association between PMS and analgesic use was statistically significant (χ²=27.09, p<0.001) (Table 6).

**Table 6:** Association between PMS and analgesic use among female medical students Values are presented as number (%). Associations between categorical variables were assessed using the Pearson Chi-square (χ²) test (χ²=27.09, p<0.001). PMS: premenstrual syndrome

PMS status	Analgesics used (number (%))	Analgesics not used (number (%))	Total (number (%))	χ²	p-value
Present	67 (35.6)	121 (64.4)	188 (75.2)	27.09	<0.001
Absent	2 (3.2)	60 (96.8)	62 (24.8)		
Total	69 (27.6)	181 (72.4)	250 (100.0)		

Multivariate logistic regression analysis revealed that cramps (AOR=2.76, p<0.001), stress (AOR=2.38, p=0.001), lack of concentration (AOR=2.45, p=0.002), mood swings (AOR=2.11, p=0.009), and inadequate sleep (AOR=1.91, p=0.017) were significant predictors of reduced work productivity. Junk food consumption did not show a statistically significant association (Table 7).

**Table 7:** Logistic regression analysis for predictors of reduced work productivity Multivariable logistic regression analysis showing independent predictors of reduced productivity among female medical students with PMS. AORs with 95% CI were obtained using multivariable logistic regression. Statistical significance was assessed using the Wald test. AOR: adjusted odds ratio, CI: confidence intervals

Variable	AOR	95% CI	p-value
Stress	2.38	1.42-3.99	0.001
Inadequate sleep	1.91	1.12-3.24	0.017
Cramps	2.76	1.60-4.76	<0.001
Mood swings	2.11	1.19-3.72	0.009
Lack of concentration	2.45	1.38-4.35	0.002
Junk food consumption	1.29	0.74-2.24	0.358

## Discussion

Premenstrual syndrome (PMS) is a common cyclical disorder occurring during the luteal phase of the menstrual cycle and typically resolves with the onset of menstruation. It is largely attributed to hormonal fluctuations, particularly involving estrogen and progesterone, and has significant implications on the physical, emotional, and social well-being of affected individuals [8-10]. Cultural stigma and lack of awareness in certain regions, including parts of India, may further limit health-seeking behavior among women.

In the present study, the prevalence of PMS was 188 (75.2%), which is comparable to findings from other studies, although slightly lower than the 86% prevalence reported among college-going girls in Karnataka [9]. Previous literature from India and other regions also reports a high prevalence of PMS, highlighting its widespread nature [2,9]. The most common somatic symptom observed in our study was cramps (153, 61.2%), followed by bloating (107, 42.8%) and breast tenderness (59, 23.6%). Among psychological symptoms, mood swings (191, 76.4%) were the most frequently reported, followed by overthinking and anger issues. These findings are consistent with existing literature, which identifies both affective and somatic symptoms as key components of PMS [5,11].

PMS was found to have a significant impact on productivity and daily functioning. In the present study, 147 (58.8%) participants reported reduced overall productivity, while 144 (57.6%) participants experienced decreased academic or work performance. Furthermore, a statistically significant association was observed between PMS and reduced productivity (χ²=67.58, p<0.001). Similar findings have been reported in previous studies, demonstrating that PMS is associated with impaired functional capacity, decreased efficiency, and reduced quality of life [4,12]. Published research reports productivity losses of up to 39.6% in women with PMS compared to 18.1% in those without PMS, contributing to both absenteeism and presenteeism [13].

The present study also identified significant associations between PMS and certain lifestyle factors. Mental stress was reported by 124 (66.0%) participants with PMS compared with 25 (40.3%) participants without PMS (χ²=12.80, p<0.001), highlighting the bidirectional relationship between psychological stress and symptom severity. Similarly, junk food consumption was reported by 139 (73.9%) participants with PMS and 38 (61.3%) participants without PMS; however, the association was not statistically significant (χ²=3.69, p=0.055). Analgesic use was reported by 67 (35.6%) participants with PMS compared with 2 (3.2%) participants without PMS (χ²=27.09, p<0.001), reflecting the burden of somatic symptoms and the need for symptomatic relief.

Multivariable logistic regression analysis revealed that cramps, mental stress, lack of concentration, mood swings, and inadequate sleep were independent predictors of reduced productivity, whereas junk food consumption was not significantly associated. These findings emphasize the multifactorial nature of PMS and its substantial impact on functional outcomes.

Premenstrual syndrome has a multifactorial etiology, and its management is primarily aimed at symptomatic relief. This includes alleviation of both physical discomfort and psychological distress through a combination of lifestyle modifications, psychological interventions, and pharmacological therapy [14,15]. Lifestyle measures such as regular physical activity, a balanced diet, adequate sleep, and stress reduction techniques (e.g., yoga and meditation) play a crucial role in symptom control [8,15].

In the present study, the most commonly adopted coping strategies included binge eating (84, 33.6%), analgesic use (69, 27.6%), and engagement in recreational activities such as watching television or movies. Pharmacological management, particularly with selective serotonin reuptake inhibitors and hormonal therapies such as combined oral contraceptives, is effective in moderate to severe cases [15]. Cognitive-behavioral therapy serves as an important adjunct by helping individuals develop coping mechanisms for emotional and behavioral symptoms [16]. Furthermore, patient education and psychosocial support play a vital role in empowering women for self-care and improving treatment adherence [17,18]. Overall, a tailored, multimodal approach is essential for effective PMS management in young women, addressing both symptom relief and functional improvement.

Strengths and limitations

Our study gives valuable insights into the prevalence and impact of premenstrual syndrome among young women, with a relatively large sample size including both somatic and affective symptoms. The use of statistical analysis, including regression models, strengthens the reliability of the findings and highlights important predictors of reduced productivity.

However, the study has certain limitations. Its cross-sectional design does not permit causal inferences, and PMS was assessed using a retrospective self-reported questionnaire rather than prospective daily symptom recording, which may have introduced recall bias and symptom misclassification. The investigator-developed questionnaire and productivity assessment were not formally validated, although they were reviewed by subject experts for content relevance and clarity. In addition, the study was conducted at a single medical university and included only female medical students aged 18-25 years; therefore, the findings may not be generalizable to women from other educational settings, age groups, or the general population. Potential confounding factors such as dietary patterns, physical activity levels, and underlying psychological conditions were not explored in detail. Finally, the study did not assess the severity grading of PMS or differentiate clearly between PMS and premenstrual dysphoric disorder, which could provide deeper clinical insights.

## Conclusions

Premenstrual syndrome was highly prevalent among female medical students in the present study, affecting three-fourths of the participants. Mood swings, anxiety, and abdominal cramps were the most frequently reported symptoms and were associated with a significant reduction in academic productivity and daily functioning. Mental stress, inadequate sleep, cramps, mood swings, and lack of concentration emerged as independent predictors of reduced productivity, highlighting the influence of both psychological and lifestyle factors on PMS.

These findings underscore the need for greater awareness of PMS among young women and educational institutions. Early identification, appropriate counselling, lifestyle modifications, and timely medical intervention may help reduce symptom severity and improve academic performance and overall quality of life.

## Appendices

Study questionnaire

Participant Information Sheet

You are invited to participate in a research study. The purpose of this study is to estimate the prevalence of premenstrual syndrome (PMS), identify associated lifestyle factors, and assess its impact on academic performance and productivity among female medical students. Your participation is entirely voluntary.

The questionnaire will take approximately 10-15 minutes to complete. You may choose not to answer any question or withdraw from the study at any time without any academic or personal consequences. Information will remain confidential and anonymous and will be used solely for research purposes. For any queries regarding this study, you may contact the Principal Investigator.

Informed Consent

I have read the participant information sheet. I voluntarily agree to participate.

Participant Signature: __________   Date: __________

Participant Details

Participant ID: ______ (to be filled by the Investigator)

Age: ____ years

Year of study: __________

Regular menstrual cycles: □ Yes □ No

Study Questionnaire

Table 8 presents the questionnaire used in the present study.

**Table 8:** Study questionnaire used to assess premenstrual syndrome, lifestyle factors, productivity, and coping strategies

Section	Question/variable	Response
B. Premenstrual symptoms (tick all symptoms experienced during the premenstrual period)	Anger issues	☐
	Anxiety	☐
	Breast tenderness	☐
	Bloating	☐
	Constipation	☐
	Cramps	☐
	Diarrhea	☐
	Depression	☐
	Headache	☐
	Lack of concentration	☐
	Mood swings	☐
	Overthinking	☐
	None	☐
C. Lifestyle habits	Junk food consumption	□ Yes □ No
	Smoking/tobacco/alcohol use	□ Yes □ No
	Caffeine intake (>2 cups/day)	□ Yes □ No
	Inadequate sleep (<7 hours/day)	□ Yes □ No
	Regular exercise/yoga	□ Yes □ No
	Mental stress	□ Yes □ No
D. Effect of PMS on productivity	Overall productivity affected	□ Yes □ No
	Academic performance affected	□ Yes □ No
	Concentration during study affected	□ Yes □ No
	Lecture attendance affected	□ Yes □ No
	Clinical postings affected	□ Yes □ No
	Routine daily activities affected	□ Yes □ No
E. Interventions used for symptom relief (tick all that apply)	Analgesics	☐
	Hot drinks	☐
	Massage	☐
	Binge eating (chocolate/food)	☐
	Watching movies/TV	☐
	Other	☐
	No treatment	☐

## References

[1] Tiranini L, Nappi RE (2022). Recent advances in understanding/management of premenstrual dysphoric disorder/premenstrual syndrome. Fac Rev.

[2] Dutta A, Sharma A (2021). Prevalence of premenstrual syndrome and premenstrual dysphoric disorder in India: a systematic review and meta-analysis. Health Promot Perspect.

[3] (2023). Management of premenstrual disorders: ACOG clinical practice guideline no. 7. Obstet Gynecol.

[4] Itriyeva K (2022). Premenstrual syndrome and premenstrual dysphoric disorder in adolescents. Curr Probl Pediatr Adolesc Health Care.

[5] Ryu A, Kim TH (2015). Premenstrual syndrome: a mini review. Maturitas.

[6] Siminiuc R, Ţurcanu D (2023). Impact of nutritional diet therapy on premenstrual syndrome. Front Nutr.

[7] Sullivan KM, Dean A, Soe MM (2009). OpenEpi: a web-based epidemiologic and statistical calculator for public health. Public Health Rep.

[8] Modzelewski S, Oracz A, Żukow X, Iłendo K, Śledzikowka Z, Waszkiewicz N (2024). Premenstrual syndrome: new insights into etiology and review of treatment methods. Front Psychiatry.

[9] Upadhyay M, Mahishale A, Kari A (2023). Prevalence of premenstrual syndrome in college going girls - a cross sectional study. Clin Epidemiol Glob Health.

[10] Abu Alwafa R, Badrasawi M, Haj Hamad R (2021). Prevalence of premenstrual syndrome and its association with psychosocial and lifestyle variables: a cross-sectional study from Palestine. BMC Womens Health.

[11] Pinkerton JV, Goje O (2026). Premenstrual syndrome (PMS). https://www.msdmanuals.com/professional/gynecology-and-obstetrics/abnormal-uterine-bleeding/premenstrual-syndrome-pms.

[12] Yonkers KA, O'Brien PM, Eriksson E (2008). Premenstrual syndrome. Lancet.

[13] Loukzadeh Z, Eslamy N, Dehghan M, Houshang Mehrparvar A (2024). The impact of premenstrual disorders on work disruptions among working women: a cross-sectional study. Int J Reprod Biomed.

[14] Pullayikudi SP, Sood A (2025). The clinical impact and management of premenstrual syndrome. Gynecol Obstet Reprod Med.

[15] Carlini SV, Lanza di Scalea T, McNally ST, Lester J, Deligiannidis KM (2022). Management of premenstrual dysphoric disorder: a scoping review. Int J Womens Health.

[16] Gudipally PR, Sharma GK (2023). Premenstrual syndrome. StatPearls [Internet].

[17] Liguori F, Saraiello E, Calella P (2023). Premenstrual syndrome and premenstrual dysphoric disorder’s impact on quality of life, and the role of physical activity. Medicina (Kaunas).

[18] Costanian C, Akiki Z, Rabah Z, Daou S, Assaad S (2018). Factors associated with premenstrual syndrome and its different symptom domains among university students in Lebanon. Int J Womens Health Wellness.

